# The prognostic impact of body mass index on female breast cancer patients in underdeveloped regions of northern China differs by menopause status and tumor molecular subtype

**DOI:** 10.1515/biol-2022-0748

**Published:** 2023-10-24

**Authors:** Lijun Ma, Ailan Liu, Jinnan Gao, Haoliang Zhao

**Affiliations:** Department of Breast Surgery, Third Hospital of Shanxi Medical University, Shanxi Bethune Hospital, Shanxi Academy of Medical Sciences, Tongji Shanxi Hospital, Taiyuan, 030002, China; Department of Clinical Laboratory, Second Hospital of Shanxi Medical University, Taiyuan, 030001, China; Department of General Surgery, Third Hospital of Shanxi Medical University, Shanxi Bethune Hospital, Shanxi Academy of Medical Sciences, Tongji Shanxi Hospital, Taiyuan, 030032, China

**Keywords:** body mass index, breast neoplasms, prognosis, survival

## Abstract

There is growing evidence that higher body mass index (BMI) is associated with lower survival in breast cancer patients. The aim of this study was to investigate whether there is an association between body mass index (BMI) at breast cancer diagnosis and breast cancer prognosis and whether this association is dependent on menopausal status and tumor subtype in a less developed population in northern China. We collected 1,225 patients with primary invasive cancer in stage I-IIIC for retrospective analysis from October 2010 to December 2020. We used Kaplan–Meier and Cox regression analyses and estimated the relationship between baseline BMI and breast cancer-specific survival (BCSS). Next, we further evaluated whether the effect of BMI on breast cancer prognosis differed by menopausal status and tumor subtype. We found that death rate and prognosis were worse for patients with BMI ≥ 24, more than four positive lymph nodes, and triple negative status. Interestingly, BMI played a different prognostic role depending on tumor subtype and menopausal status. For premenopausal women, patients with BMI ≥ 24 had significantly lower BCSS compared to those with BMI < 24 in human epidermal growth factor receptor 2 (HER2) overexpression (HR: 4.305, *p* = 0.004) and triple negative subtypes (HR: 1.775, *p* = 0.048). By contrast, there was no association between BMI ≥ 24 and higher death regardless of tumor subtype in post-menopausal patients (*p* > 0.05). BMI influences breast cancer outcome depending on tumor subtype and menopause. BMI ≥ 24 might be a risk factor for BCSS, particularly in premenopausal women with HER2 overexpression or triple negative subtype. In contrast, BMI ≥ 24 was not associated with higher death regardless of tumor subtype in post-menopausal patients.

## 1 Introduction

In 2020, there were an estimated 2.3 million cases, with cancer surpassing cancer and becoming the most common type of cancer among women. Globally, 685,000 people die of cancer each year due to this disease, according to GLOBOCAN 2020 [1]. The incidence and mortality rates of cancer are high in Asia. Although cancer mortality was decreasing, its incidence was increasing, particularly among females and younger people [2]. As a low-income developing country, China was experiencing an increasing cancer burden in the breast [3].

Obesity, a modifiable characteristic, is another major global health concern. Although controversial, we considered that obesity and underweight may negatively affect breast cancer outcomes. Studies have reported that obesity is associated with an increased risk and a negative outcome for breast cancer [4,5]. However, research has shown that underweight is associated with a poor prognosis [6,7]. Although the exact mechanisms are not well understood, obesity-associated inflammation may contribute to increased breast cancer risk as well as poor breast cancer outcomes in obese women [5].

The most widely used measure of obesity is the body mass index (BMI), calculated by multiplying the height and weight of an individual. Many studies have investigated the influence of BMI on breast cancer outcomes, but few have focused on Asian populations, especially in China. Depending on tumor subtype or menopausal status, the role of BMI in prognosis is controversial. Heterogeneity of the study population, menopausal status, and tumor subtypes may have influenced this result. In this study, we aimed to retrospectively investigate whether baseline BMI influenced prognosis based on tumor type and menopausal status in patients with breast cancer. This was done in a cohort study of women with primary breast cancer who were treated at a facility in a less-developed part of China.

## 2 Material and methods

### 2.1 Study cohort and patient selection

In this retrospective cohort study, we analyzed all patients with primary invasive breast cancer (*n* = 1,872) diagnosed (and histologically confirmed) at Shanxi Bethune Hospital between October 2010 and December 2020, with stages ranging from I to IIIC. Written informed consent was obtained from all patients during the research process, and each study was approved by the institutional review board. We extracted baseline (date of diagnosis) data from their medical records, including socio-demographic variables (such as height, weight, age, sex, date of diagnosis and date of death), clinicopathologic characteristics (such as tumor size, pathological nodal status, histopathological type and grade, estrogen receptor (ER), progesterone receptor (PR), human epidermal growth factor receptor 2 (HER2), and Ki-67, tumor subtype), clinical information (such as menstrual status, disease stage, breast cancer-specific mortality, follow-up time, and vital status).

**Figure 1:**
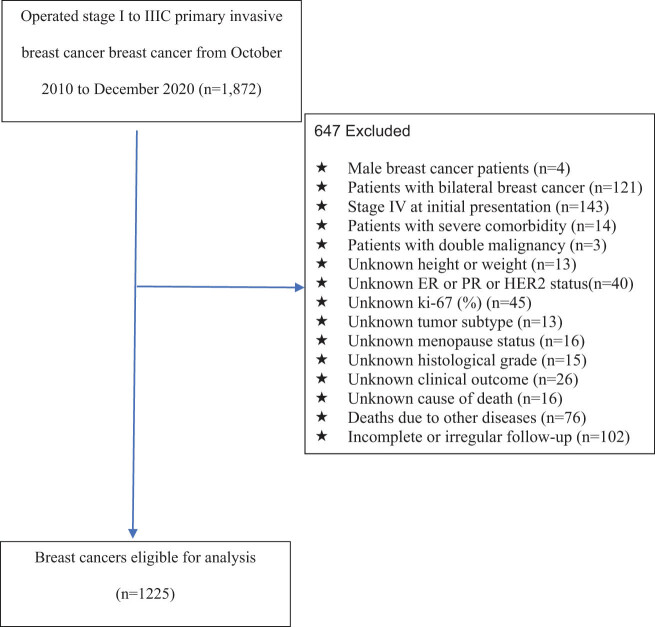
Flow diagram of the selection of the eligible population.


As a first step, we excluded patients with bilateral breast cancer (*n* = 121), male breast cancer (*n* = 4), stage IV breast cancer at initial presentation (*n* = 143), severe comorbidities (*n* = 14), and double malignancy (*n* = 3). Then, we excluded patients with incomplete information on ER, PR and HER2 receptor statuses (*n* = 40), patients with incomplete height and weight information (*n* = 13), patients with unknown ki-67(%) (*n* = 45), patients with unknown tumor subtypes (*n* = 13), patients with uncertain menopausal status (*n* = 16), and patients with unclear histological grades (*n* = 15). Further, we eliminated patients with unclear clinical outcomes (*n* = 26), deaths from other diseases (*n* = 76), and incomplete or irregular follow-up (*n* = 102). Figure 1 shows the flow diagram of patient selection. In total, 1,225 patients were analyzed. Patients were checked every 3 months for the first 2 years, every 6 months for 2–5 years, and then once a year for 5–10 years. We followed every patient until death, death from any cause, or until 31 December 2020. After the last day of follow-up, patients who were alive were censored.

**Informed consent:** Informed consent has been obtained from all individuals included in this study.
**Ethical approval:** The research related to human use has been complied with all the relevant national regulations, institutional policies and in accordance with the tenets of the Helsinki Declaration, and has been approved by the Medical Ethics Review Committee at Shanxi Bethune Hospital (No. YXLL-KY-2021-010).

### 2.2 Measurement of baseline BMI

The World Health Organization (WHO) defines BMI as weight in kilograms divided by height in meters (kg/m^2^), which was measured by a study nurse. WHO defines overweight as a BMI between 25 and 29.9 kg/m^2^, while obesity is defined as a BMI of 30 kg/m^2^ or higher [8]. The Obesity Working Group of the China Office of the International Society for Life Sciences has determined the obesity limit for Chinese adults to judge the degree of overweight and obesity based on large-scale measurements. The Guidelines for the Prevention and Treatment of Overweight and Obesity in Chinese Adults recommend that a BMI of 24.0–27.9 be overweight and a BMI of 28 or greater be obese. Since Asian women tend to be thinner than Western women, we used BMI 24 as the dividing point for the analysis of prognoses for breast cancer patients at BMI ≥ 24 and BMI < 24.

### 2.3 Analysis of breast cancer subtype

From the pathology report, we obtained the clinical data regarding tumor characteristics. According to American Society of Clinical Oncology/College of American Pathologists Guideline Recommendations [9,10,11], immunohistochemical (IHC) analysis was used to assess the status of ER, PR, HER2, and Ki67, and fluorescence in situ hybridization (FISH) was used for patients with HER2 IHC grade 2. Nuclear staining of the tumor cells revealed the presence of ER and PR, while the staining of the tumor cell membrane showed the presence of HER2. In tumor cells, ER and PR expression was positive when ≥1% of stained cells were present and negative when <1% stained cells were present. When ER and PR are present, the hormone receptor (HR) is defined as positive. When HER2 IHC was grade 3 or grade 2 with FISH positive, HER2 status was defined as positive. Also, it was determined that the Ki-67 index would be high if the number of tumor cells was greater than 14% in the tissue. St Gallen’s subtype classification scheme [12] divided all breast cancer patients into five subgroups: Luminal A: HR+, HER2− and Ki-67 levels are low; Luminal B (HER2−): ER+ and HER2− are required, along with at least one of the following: Ki-67 high or PR ‘negative or low’; Luminal B (HER2+): ER+, HER2+, regardless of Ki-67 and PR levels; HER2-overexpression: ER−, PR−, HER2+; triple-negative: ER−, PR−, HER2−.

### 2.4 Identification of menstrual status

Menopausal women are those without menstruation for 12 consecutive months or who have had their bilateral ovaries removed.

### 2.5 Survival data collection

Follow-up, including hospital visits and telephone or email interviews, begins on the first day after surgery, every 3 months for the first two years, every 6 months for the next 2 to 5 years, and annually for the next 5 years. The follow-up ended on December 31, 2020. The date and cause of death of patients were obtained from hospital death certificates or the patient’s family. All non-breast cancer causes of death have been censored at the date of death. In our study, time to breast cancer death was the primary outcome. Therefore, breast cancer-specific survival (BCSS) was the primary endpoint.

### 2.6 Statistical analysis

Descriptive statistics were used to summarize baseline characteristics. Categorical variables were reported as percentages and frequency in descriptive statistics. The normal distribution variables were expressed by mean and standard deviation, while the abnormal distribution variables were expressed by median and interquartile range. Chi-square test was used to compare the differences in age, menstrual status, surgical method, histological grade, tumor size, lymph node status, ER/PR/HER2/Ki67 status, tumor subtypes, and other clinicopathologic characteristics among BMI groups.

Breast cancer specific survival (BCSS) was assessed as the survival endpoint. BCSS was defined as the time from surgery until the date of death from breast cancer. The Kaplan-Meier method was used to estimate BCSS functions. In order to determine whether survival curves differed between the groups, log-lank tests were conducted. In both univariate and multivariate analyses, Cox’s proportional hazards regression was used to estimate the hazard ratio (HR) and 95% confidence interval (CI). In the multivariable analysis, age, BMI, number of positive lymph nodes, ER, PR, KI-67, and tumor subtypes were considered.

We used SPSS 22 (IBM Corp., Armonk, USA) to conduct all statistical analyses. All tests were two-sided and a *P* < 0.05 significance level was used.

## 3 Results

### 3.1 Patients’ clinicopathological characteristics at baseline according to BMI

In Table 1, we summarized the baseline characteristics. Most patients (96%) presented with grade II or III, and most (86.45%) were over 40. According to the BMI cut-off point for Chinese adults proposed by the China Obesity Working Group (WGOC) of the International Association of Life Sciences, we divided 1,225 patients into two subgroups: BMI < 24 kg/m^2^ group of 506 individuals (41.3%) and BMI ≥ 24 kg/m^2^ group of 719 individuals (58.7%). The median age at diagnosis was significantly greater in patients with BMI ≥ 24 than in patients with BMI < 24 (*p* < 0.001). The proportion of postmenopausal patients in the BMI ≥ 24 group was significantly higher than that in the BMI < 24 group (*p* < 0.001). BMI < 24 patients had high frequencies of HER2 expression (*p* = 0.020) compared with BMI ≥ 24 patients. However, there were no significant differences in surgical methods, histological grades, number of lymph nodes, tumor size, ER, PR, ki-67, and molecular subtypes between BMI ≥ 24 and BMI < 24 groups (*p* > 0.05).

**Table 1:** Baseline characteristics of 1,225 Chinese female patients with breast cancer according to BMI

Characteristics	Total		BMI < 24		BMI ≥ 24		*P* value
	*N*	%	*N*	%	*N*	%	
Total	1,225		506		719		
**Age at diagnosis (year)**							
<40	166	13.55	92	18.18	74	10.29	<0.001
≥40	1059	86.45	414	81.82	645	89.71	
**Menstrual status at diagnosis**							
Pre-menopausal	640	52.24	300	59.29	340	47.29	<0.001
Post-menopausal	585	47.76	206	40.71	379	52.71	
**Surgery**							
Conserving	537	43.84	211	41.70	326	45.34	0.206
Mastectomy	688	56.16	295	58.30	393	54.66	
**Histological grade**							
Grade 1	48	3.92	23	4.55	25	3.48	0.257
Grade 2	768	62.69	326	64.43	442	61.47	
Grade 3	409	33.39	157	31.03	252	35.05	
**Tumor size (cm)**							
≤2	539	44.00	241	47.63	298	41.45	0.091
2–5	585	47.76	224	44.27	361	43.95	
>5	101	8.24	41	8.10	60	8.34	
**Number of positive lymph nodes**							
0	366	29.88	150	29.64	216	30.04	0.813
1–3	409	33.39	165	32.61	244	33.94	
≥4	450	33.06	191	37.75	259	36.02	
**ER status**							
Positive	813	66.37	329	65.02	484	67.32	0.402
Negative	412	33.63	177	34.98	235	32.68	
**PR status**							
Positive	675	55.10	275	54.35	400	55.63	0.656
Negative	550	44.90	231	45.65	319	44.37	
**HER2 status**							
Positive	233	19.02	112	22.13	121	16.83	0.020
Negative	992	80.98	394	77.87	598	83.17	
**Ki-67 (%)**							
≤14	286	23.35	111	21.94	175	24.34	0.328
>14	939	76.65	395	78.06	544	75.66	
**Subtype**							
Luminal-A	90	7.35	35	6.92	55	7.65	0.632
Luminal-B (HER2−)	591	48.24	236	46.64	355	49.37	
Luminal-B (HER2+)	116	9.47	55	10.87	61	8.48	
HER2 overexpression	239	19.51	101	19.96	138	19.19	
Triple negative	189	15.43	79	15.61	110	15.30	

**Abbreviations:** BMI, body mass index; ER, estrogen receptor; PR, progesterone receptor; HER2, human epidermal growth factor receptor 2.

### 3.2 Univariate and multivariate analyses

To further observe the difference, we showed the univariate and multivariate analysis results. In univariate analysis, BMI, number of lymph nodes, ER and PR status, Ki-67 status, and tumor subtype were statistically significant predictors of prognosis. Indeed, breast cancer patients with BMI ≥ 24 (HR: 1.881), four or more positive lymph nodes (HR: 2.247), Ki-67 ≥ 14% (HR: 1.797), or triple-negative (HR: 2.332) had a significantly higher mortality rate. In contrast, patients with ER positive (HR: 0.435), PR positive (HR: 0.467), and Luminal-A positive (HR: 0.137) had significantly better survival. According to the study, women with BMI ≥ 24 kg/m^2^ at diagnosis had 88.1% higher mortality than women with normal BMI (<24 kg/m^2^).

According to clinical experience and univariate analysis results, six variables (age, number of positive lymph nodes, BMI, ER, PR, ki-67, and tumor subtype) were included in multivariate analysis. The results of multivariate analyses indicated that BMI could be regarded as a statistically significant independent factor (HR, 2.130; 95% CI, 1.532–2.963; *p* < 0.001). ER, PR, KI-67, and tumor subtype were significant factors in univariate analyses, but lost significance in multivariate analyses. Correspondingly, breast cancer patients with BMI ≥ 24 (HR: 2.130), more than 4 positive lymph nodes (HR: 1.604), and triple negative (HR: 2.408) had a significantly higher probability of death. By contrast, patients with Luminal-A (HR: 0.201) had significantly improved survival. We found that women whose BMI at diagnosis was above 24 kg/m^2^ faced a substantial 1.30 times increased risk of death compared with women whose BMI was normal (<24 kg/m^2^).

Overall, patients with BMI ≥24, ≥4 positive lymph nodes and triple negative had the highest mortality and the worst prognosis, while patients with BMI < 24, negative lymph nodes, and Luminal-A had significantly better survival and the most favorable prognosis.

### 3.3 Prognostic implication of baseline BMI

Figure 1 shows that 1,225 women with breast cancer were enrolled in the present study, with a mean age of 52.03 ± 11.80 years at diagnosis. The median follow-up time after breast cancer surgery was 41.07 months (27.82–55.27), and the longest follow-up time was 101 months. During this period, 171 breast cancer-related deaths were observed. In addition, 76 deaths observed due to other diseases were excluded by exclusion criteria.

**Figure 2:**
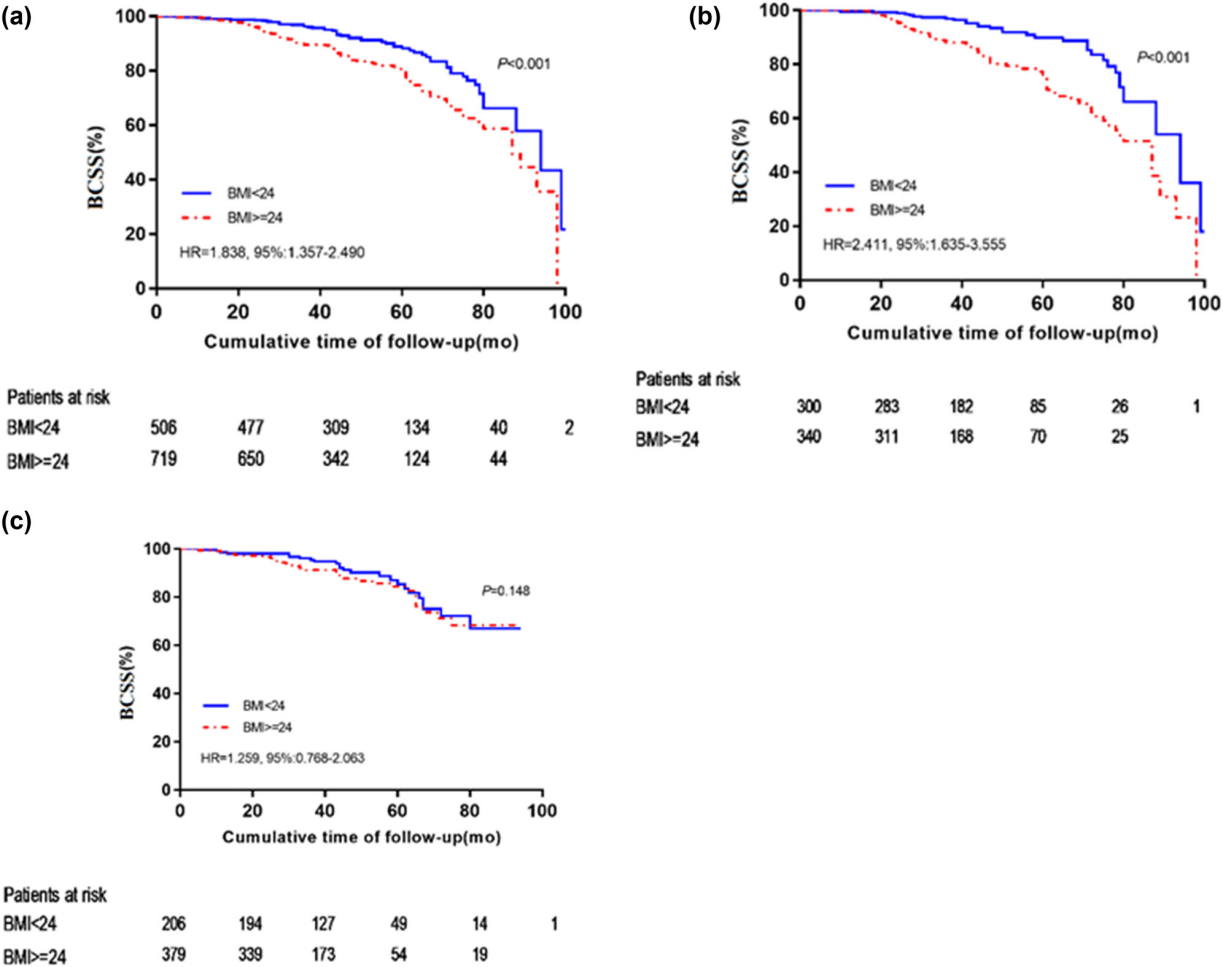
Kaplan-Meier curves show the BCSS according to baseline BMI in (a) all 1,225 patients, (b) 640 premenopausal patients, and (c) 585 postmenopausal patients (c).

Cox proportional hazard model showed that the BMI ≥ 24 group had significantly poorer prognosis than the BMI < 24 group (HR, 1.881; 95% CI, 1.364–2.593; *p* < 0.001) (Table 2). Kaplan–Meier analysis showed that the mortality rate of patients with BMI ≥ 24 was higher than that of patients with BMI < 24. The survival rate of 41.07 months for breast cancer patients in the BMI < 24 group and BMI ≥ 24 group was 96.4% and 91.5%, respectively, and the difference was statistically significant [HR, 1.838; 95% CI, 1.357–2.490; *p* < 0.001 (Figure 2a).

**Table 2:** Univariable and multivariable Cox regression analysis for prognostic factors of breast cancer related to breast cancer-specific death

Variable	Alive	Death	Univariate			Multivariate		
			HR	95%CI	*p*	HR	95%CI	*p*
No. of participants	1,054	171						
**Age at diagnosis (year)**								
<40	134(12.7)	32(18.7)	1			1		
≥40	920(87.3)	139(81.3)	0.787	0.531–1.168	0.234	0.674	0.449–1.012	0.057
BMI								
<24			1			1		
≥24			1.881	1.364–2.593	<0.001	2.130	1.532–2.963	<0.001
**Menstrual status at diagnosis**								
Pre-menopausal	526(49.9)	92(53.8)	1					
Post-menopausal	528(50.1)	79(46.2)	0.838	0.614–1.143	0.265			
**Surgery**								
Conserving	472(44.8)	65(38.0)	1					
Mastectomy	582(55.2)	106(62)	1.206	0.884–1.646	0.237			
**Histological grade**								
Grade 1	45(4.3)	3(1.8)	1					
Grade 2	663(62.9)	105(61.4)	1.735	0.549–5.482	0.348			
Grade 3	346(32.8)	63(36.8)	2.281	0.715–7.274	0.163			
**Tumor size (cm)**								
≤2	469(44.5)	70(40.8)	1					
2–5	500(47.4)	85(49.7)	0.932	0.539–1.611	0.800			
>5	85(8.1)	16(9.4)	0.903	0.528–1.545	0.710			
**Number of positive lymph nodes**								
0	334(31.7)	32(18.7)	1			1		
1–3	353(33.5)	56(32.7)	1.618	1.047–2.500	0.030	1.214	0.775–1.903	0.397
≥4	367(34.8)	83(48.5)	2.247	1.494–3.381	<0.001	1.604	1.053–2.445	0.028
**ER status**								
Positive	314(29.8)	98(57.3)	1			1		
Negative	740(70.2)	73(42.7)	0.435	0.321–0.591	<0.001	0.984	0.481–2.011	0.964
**PR status**								
Positive	440(41.7)	110(64.3)	1			1		
Negative	614(58.3)	61(35.7)	0.467	0.341–0.639	<0.001	0.895	0.520–1.543	0.691
**HER2 status**								
Positive	849(80.6)	143(83.6)	1					
Negative	205(19.4)	28(16.4)	0.909	0.606–1.363	0.644			
**ki-67 (%)**								
≤14	259(24.6)	27(15.8)	1			1		
>14	795(75.4)	144(84.2)	1.797	1.191–2.712	0.005	1.161	0.736–1.833	0.520
**Subtype**								
Luminal-A	88(8.3)	2(1.2)	1			1		
Luminal-B (HER2−)	533(50.6)	58(33.9)	0.774	0.415–1.445	0.422	0.949	0.497–1.814	0.875
Luminal-B (HER2+)	104(9.9)	12(7.0)	0.137	0.031–0.610	0.009	0.201	0.041–0.984	0.048
HER2 overexpression	203(19.3)	36(21.1)	1.209	0.629–2.324	0.570	1.313	0.570–3.025	0.523
Triple negative	126(12.0)	63(36.8)	2.332	1.257–4.326	0.007	2.408	1.030–5.628	0.042

**Abbreviations:** BMI, body mass index; ER, estrogen receptor; PR, progesterone receptor; HER2, human epidermal growth factor receptor 2; HR, hazard ratio; CI, confidence interval.

### 3.4 Prognostic implication of baseline BMI according to tumor subtype and menopausal status

Next, we assessed whether menopausal status and tumor subtype affected baseline BMI’s prognostic significance for BCSS. Figure 2 showed that BMI was not a prognosticator in post-menopausal patients [log-rank test, *p* = 0.148 (Figure 2c)], but was a highly significant prognostic factor in pre-menopausal patients [log-rank test, *p* < 0.001 (Figure 2b)] or in all patients [log-rank test, *p* < 0.001 (Figure 2a)]. The median survival time of premenopausal breast cancer patients was 41.63 months, and the survival rate was 96.3% in the BMI < 24 group and 90.0% in the BMI ≥ 24 group, with statistically significant differences (*p* < 0.001, Figure 2b). The median survival time of postmenopausal breast cancer patients was 40.07 months, and the survival rate was 92.9% in the BMI < 24 group and 95.6% in the BMI ≥ 24 group, with no statistically significant differences (*p* = 0.350, Figure 2c).

**Figure 3:**
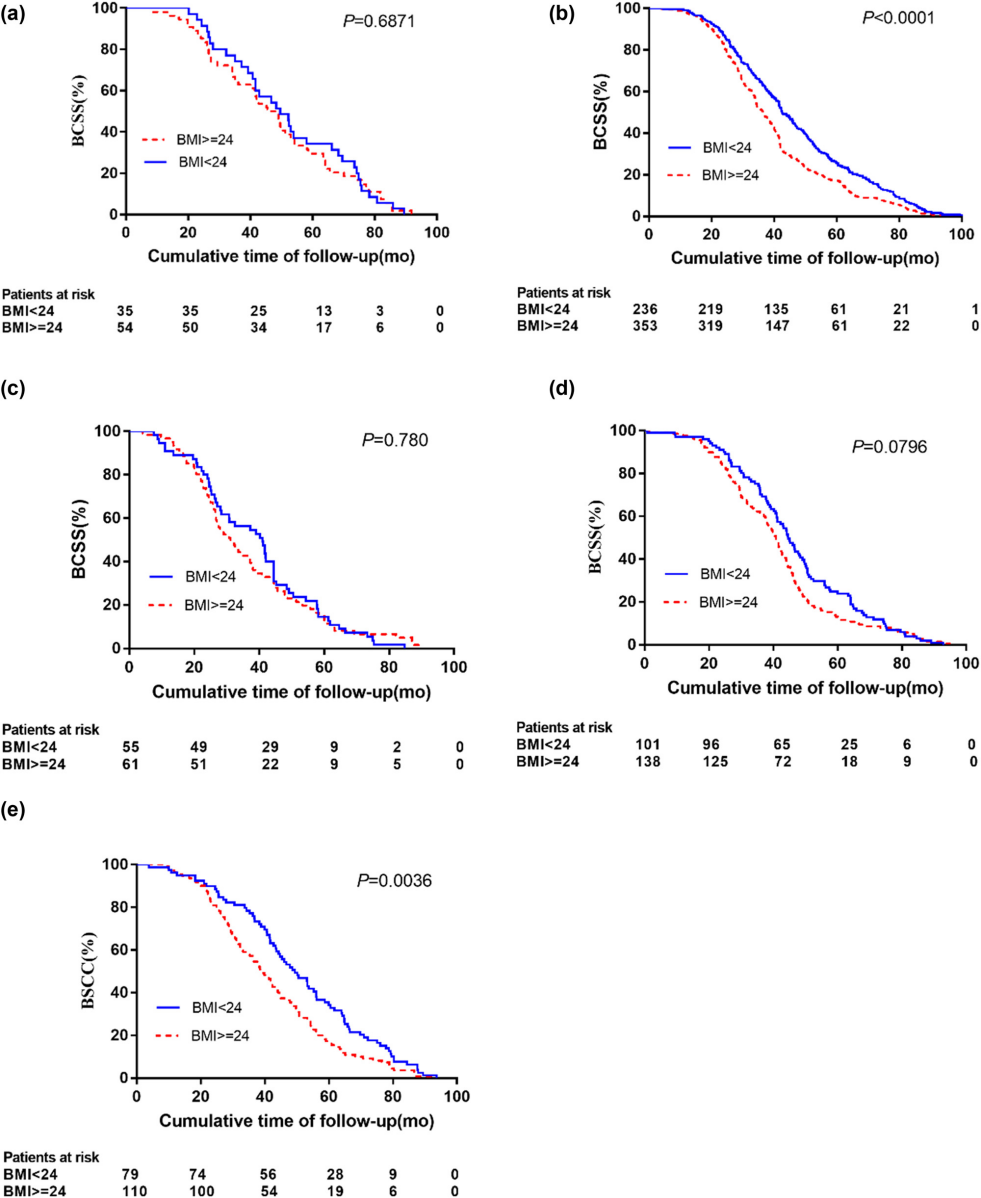
Kaplan–Meier curves show the BCSS according to baseline BMI: in (a) Luminal-A subgroup, (b) Luminal-B(HER2−) subgroup, (c) Luminal-B(HER2+) subgroup, (d) HER2 overexpression subgroup and (e) triple-negative subgroup, respectively.

In addition, tumor subtypes influence the effect of BMI on breast cancer outcomes. Luminal-A had the highest 5-year survival rate of 97.8%, followed by Luminal-B (HER2−) and Luminal-B (HER2+) (90.2 and 89.7%, respectively), and HER2 overexpression and triple-negative 5-year survival rates were the poorest (84.9 and 78.8%, respectively). Patients with BMI ≥ 24 exhibited significantly worse BCSS compared with patients with BMI < 24 in the Luminal-B (HER2−) subgroup (HR: 1.579, *p* = 0.049), in the HER2 overexpression subgroup (HR: 2.966, *p* = 0.007) or in the triple negative subgroup (HR: 2.035, *p* = 0.018) (Table 3). However, no effect of BMI on BCSS was found in patients with either Luminal-A or Luminal-B (HER2+) subgroups. As shown in Figure 3, different molecular types of breast cancer produce different KM curves. Survival rates were significantly higher in patients with a BMI < 24 than in patients with a BMI ≥ 24 in the Luminal-B (HER2−) and triple-negative groups (*p* < 0.001 and *p* = 0.036, respectively). However, there was no significant difference in BMI < 24 and BMI ≥ 24 for other types of breast cancer (*p* > 0.05).

**Table 3:** Cox’s proportional hazard regression model for breast cancer–specific survival (BCSS) by subtype and menopausal status

Subtype	Breast cancer-specific mortality								
	Overall			Pre-menopausal			Post-menopausal		
	Total	Deaths	HR	Total	Deaths	HR	Total	Deaths	HR
Luminal-A	90	2		42	1		48	1	
<24	35	0	1	19	0	1	16	0	1
≥24	55	2	53.033	23	1	105.823	32	1	38.408
*P*	*P* = 0.512			*P* = 0.095			*P* = 0.376		
Luminal-B(HER2−)	591	58		282	8		309	20	
<24	236	21	1	139	3	1	97	18	1
≥24	355	37	1.579	143	5	5.170	212	32	1.221
*P*	*P* = 0.049			*P* = 0.075			*P* = 0.661		
Luminal-B(HER2+)	116	12		74	10		42	2	
<24	55	3	1	39	3	1	26	0	1
≥24	61	9	1.653	35	7	0.850	16	2	42.807
*P*	*P* = 0.491			*P* = 0.862			*P* = 0.551		
HER2 overexpression	239	36		124	28		115	8	
<24	101	9	1	55	5	1	46	4	1
≥24	138	27	2.966	69	23	4.305	69	4	1.196
P	*P* = 0.007			*P* = 0.004			*P* = 0.985		
Triple negative	189	63		118	58		71	5	
<24	79	24	1	48	22	1	31	2	1
≥24	110	39	2.035	70	36	1.775	40	3	2.151
*P*	*P* = 0.018			*P* = 0.048			*P* = 0.372		

**Abbreviations:** BCSS, breast cancer-specific survival; HR, hazard ratio.

As shown in Table 3, BMI is associated with breast cancer risk based on molecular subtypes and menopausal status. For the premenopausal women, BMI ≥ 24 patients had significantly lower BCSS in HER2 overexpression (HR: 4.305, *p* = 0.004) and triple negative subtypes (HR: 1.775, *p* = 0.048) when compared with BMI < 24 patients. By contrast, BMI ≥ 24 was not associated with higher death regardless of tumor subtype in post-menopausal patients (*p* > 0.05).

**Figure 4:**
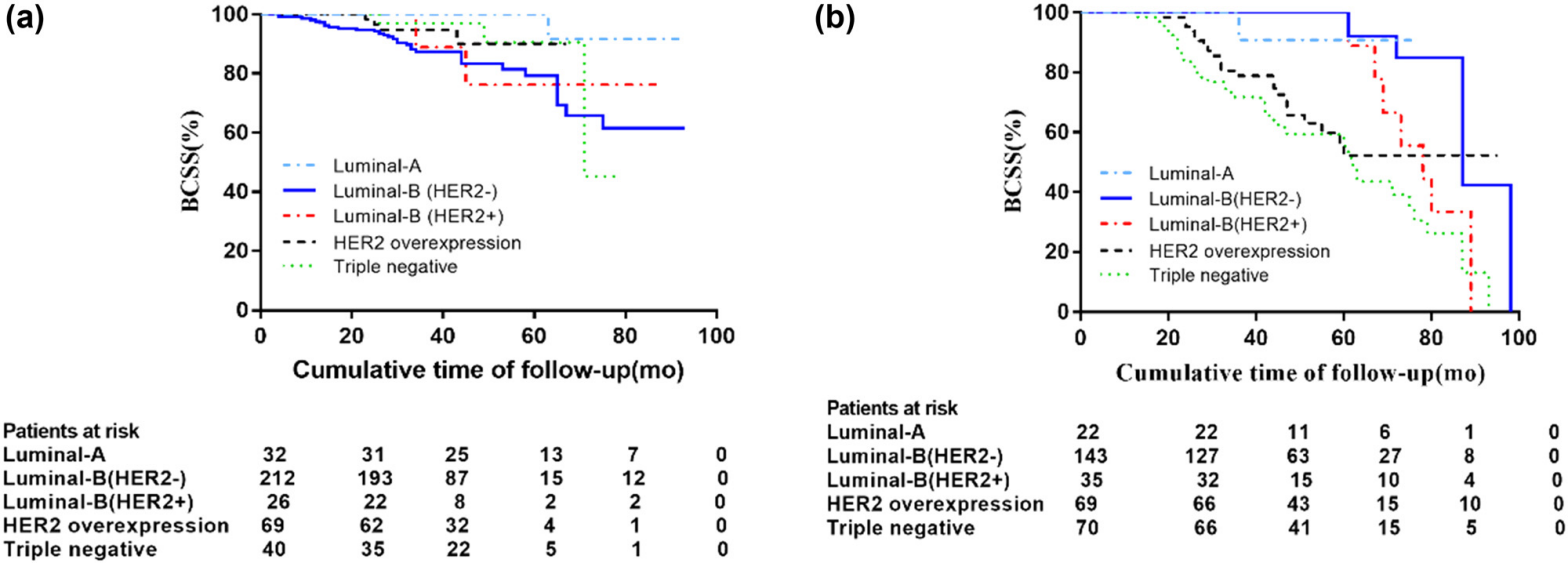
Prognostic role of tumor subtype in BMI ≥ 24 patients stratified by menopause status: (a) pre-menopausal, (b) post-menopausal.

Figure 4 showed the prognostic role of tumor subtype in BMI ≥ 24 patients stratified by menopause status. Premenopausal breast cancer patients with BMI ≥ 24 had the highest risk of death if they had triple-negative or HER2-overexpressed cancer (Figure 4a). For postmenopausal breast cancer patients with BMI ≥ 24, the risk of death from Luminal-B (HER2-negative) was highest (Figure 4b). However, these differences were not statistically significant.

## 4 Discussion

There is no doubt that obesity is a serious public health issue, and the prevalence of obesity is continuing to rise. Obesity, although controversial, has been linked with breast cancer development and mortality in numerous studies [13,14,15,16]. A recent review, [17] including 82 follow-up studies, showed that whenever BMI was determined, obesity was associated with lower overall survival and breast cancer-specific survival in premenopausal and postmenopausal women. However, most of these findings were derived from studies conducted on Western populations. It is unclear whether obesity is associated with breast cancer outcomes and mortality in Asian patients, especially in China. Interestingly, Asian and Western populations display very different types and patterns of obesity. Furthermore, due to limitations in the effectiveness of BMI as a measure of adiposity and variation in the types and patterns of obesity across populations, estimates of the risk of breast cancer prognosis associated with higher BMI have been inconsistent. Therefore, considering that Asian women are thinner than Western women, we chose the WGOC BMI cutoff of 24 rather than the WHO-recommended 25 to study the relationship between BMI and breast cancer prognosis in less developed areas of China.

In this study, results showed that the following: (1) BMI was a statistically significant independent prognostic factor for female breast cancer patients in both univariate and multivariate analyses. (2) ER, PR, ki-67, the number of positive lymph nodes, and tumor subtypes were significant factors affecting the prognosis of female breast cancer patients in univariate analysis, but they did not have significance in multivariate analysis. (3) There was a variable predictive effect associated with BMI among women with breast cancer depending on their tumor subtype and whether they were menopausal or not. (4) Patients with BMI ≥24, ≥4 positive lymph nodes, and triple-negative status had the highest mortality rate and the lowest prognosis, while those with BMI < 24, negative lymph nodes, and Luminal-A had significantly better survival and the most favorable prognosis. (5) BMI ≥ 24 might be a risk factor for BCSS, especially in premenopausal women with HER2 overexpression or triple negative subtype.

When classified according to BMI, it was found that women with higher BMI were more likely to be older, postmenopausal, and to have HER2-positive tumors. Many previous studies have confirmed these findings. A previous study demonstrated that elderly women were significantly less likely to survive than middle-aged women [15]. Other studies [18,19] have shown that postmenopausal women with a high BMI may have poorer clinical outcomes than those with a low BMI. However, in our study, the two groups of patients with BMI < 24 and BMI ≥ 24 did not differ significantly in histological grade, tumor size, and number of lymph nodes. These findings are inconsistent with many previous studies, which have shown that high BMI is related to more aggressive tumors and poorer prognosis. Possibly, our study was not able to reach the same conclusion because of the limited number of patients or because women who receive regular mammograms at age >40 years are more likely to be diagnosed earlier.

Previous studies have shown that obesity is more strongly associated with a poorer prognosis for breast cancer, with high BMI being an independent adverse prognostic factor. In our univariate analysis, BMI, number of lymph nodes, ER and PR status, Ki-67 status, and tumor subtype were statistically significant predictors of prognosis. Significantly, multivariate analysis showed that BMI remained an independent negative prognostic factor (HR, 2.130; 95% CI, 1.532–2.963; *p* < 0.001), while other factors lost their significance. In addition, Kaplan–Meier analysis further showed that patients with BMI ≥ 24 had higher mortality than those with BMI < 24, and the survival rates of breast cancer patients in BMI < 24 and BMI ≥ 24 groups were 96.4 and 91.5% at 41.07 months, respectively, with statistically significant differences (HR, 1.838; 95% CI, 1.357–2.490; *p* < 0.001). Our findings are consistent with the current majority of literature [12,14,15,16,17], some based on Western populations [20,21] and others based on Asian populations such as Japan [6], Korea [22,23,24] and China [25], that overweight and obesity are associated with poorer survival of breast cancer patients. Comparing these literatures, we can find that although different study populations come to the same conclusion, it cannot be ignored that the BMI cut-off values of these studies varied by the study population. An investigation [26] by Cossrow Nicole and Falkner Bonita in the United States analyzed the reasons for this phenomenon, suggesting that racial/ethnic differences in environmental factors such as lifestyle behavior and economic status may be part of the explanation for racial differences in obesity-related disease and disease outcomes, and differences in genetic/molecular factors may be other causes, but the mechanisms associated with this phenomenon are unclear. According to Goel et al. [27] Asians and Hispanics have lower breast cancer death rates than whites, and importantly, BMI is an effective mediator of differences between blacks and Asians.

Within limited statistical power, our study found that higher BMI had a significant effect on breast cancer-specific death in premenopausal patients [HR = 2.411; 95% CI: 1.635–3.555 for BMI ≥ 24 kg/m^2^, *p* < 0.001 (Figure 2c)], while higher BMI had no significant effect on breast cancer-specific death in postmenopausal women [HR = 1.259; 95% CI: 0.768–2.063 for BMI ≥ 24, *p* = 0.148 (Figure 2b)]. Moreover, our study showed that premenopausal breast cancer patients had a 41.63-month median survival and that the survival rates for the BMI < 24 group and BMI ≥ 24 group were 96.3 and 90.0%, respectively, with a statistically significant difference (*p* < 0.001, Figure 2b). A median survival time of 40.07 months was observed in postmenopausal breast cancer patients, and the survival rate was 92.9% in the BMI < 24 group and 95.6% in the BMI ≥ 24 group, with no statistically significant difference (*p* = 0.350, Figure 2c). Our above results demonstrated that the prognostic effect of BMI is strongly influenced by menopausal status, with higher BMI significantly associated with lower BCSS in premenopausal women. Our results are consistent with several previous observational studies [28,29,30,31] in premenopausal women or younger, which have shown that elevated BMI leads to poor overall survival. The effect of obesity on all-cause death and mortality from breast cancer was greater among premenopausal women than among postmenopausal women, according to a meta-analysis [32] of 43 studies. According to a study by Kawai et al. [6] premenopausal breast cancer patients with higher BMIs had a greater chance of dying from all causes after adjusting for clinical and known factors associated with the risk of death in breast cancer patients.

However, there are also some studies that are inconsistent with our conclusions. Currently, research conclusions on whether menopausal status influences BMI-related breast cancer prognosis is still debated. Regardless of menopausal status, some researchers have found that a high BMI is associated with a poorer breast cancer prognosis. Others, however, suggest that menopause plays a role in the association between a high BMI and a poorer prognosis for breast cancer. Notably, female breast cancer survival is not consistently associated with increased BMI. Some studies [33,34,35] show a significant association only in postmenopausal females, while others [6,21,28,29] show a significant association only among premenopausal females. The study by Kim et al. [24] found that obesity had a negative effect on postmenopausal survival, but not on premenopausal survival. Considering this, it would appear advisable to evaluate these findings in Asian populations in the future and compare them with Western populations. Interestingly, a recent systematic literature review [17] pointed out that pre- and postmenopausal women with obesity appear to have higher breast cancer mortality rates, and that summary risk estimates for premenopausal breast cancer appear to be stronger than for postmenopausal breast cancer. This literature further illustrates that although controversial, most of the findings are consistent with our conclusions.

These variable results can be explained by several factors: One reason for the difference between our results and previous results could be explained by differences in experimental design, adjuvant therapy, particular population, or ethnicity [14]. An explanation for the association between high BMI and poor prognosis in premenopausal breast cancer patients is that elevated local estrogen levels in the adipose tissue surrounding the tumor promote tumor growth. A second possible explanation is that high BMI may affect tumor proliferation and prognosis through hyperinsulinemia, insulin resistance, insulin-like growth factor, leptin, and other pathways [36–39]. Another more plausible explanation is that obesity-associated chronic inflammation may promote cancer angiogenesis and progression by upregulating inflammatory cytokines [5].

Another significant finding of the study is that in less developed areas of northern China, BMI affects female breast cancer outcomes depending on tumor subtype. As in other studies [33,40–42], our present study found that TNBC and HER2-overexpressed tumors had lower BCSS at 5 years (78.8 and 84.9%, respectively) compared to patients with luminal tumors and that the 5-year BCSS rate was significantly higher in Luminal-A patients (97.8%) compared with Luminal-B (HER2−) and Luminal-B (HER2+) patients (90.2 and 89.7%, respectively). Despite the limited statistical power, the present study reported significantly lower BCSS in BMI ≥ 24 patients for Luminal-B (HER2−) or triple-negative subgroups compared to BMI < 24 breast cancer patients. Interestingly, the relationship between obesity and TNBC prognosis is controversial, with some studies consistent with ours showing that women with triple-negative tumors have a poorer prognosis if their BMI is higher, but others disagree. It will take further studies to confirm this association and clarify the possible molecular mechanisms associated with obesity/overweight and TNBC survival. Considering the fact that the effect of BMI on breast tumor outcome varies by menopausal status and tumor subtype, we next include menopausal status and tumor subtype to further explore the prognostic effect of BMI on breast tumor, so as to better identify high-risk women with poor prognosis in less developed areas of China. For premenopausal women, BCSS was significantly lower in patients with BMI ≥ 24 in HER2 overexpression (HR: 4.305, *p* = 0.004) and triple-negative (HR:1.775, *p* = 0.048) than in patients with BMI < 24. By contrast, BMI ≥ 24, regardless of tumor subtype, was not associated with higher death in post-menopausal patients (*p* > 0.05). For premenopausal breast tumor patients with a BMI ≥ 24, triple-negative and HER2-overexpressing patients had the highest mortality risk, whereas for postmenopausal breast tumor patients with a BMI ≥ 24, Luminal-B (HER2-negative) patients had the highest risk of death.

Here are some limitations of this study: (1) The study was conducted in a single hospital in an underdeveloped area of northern China and all the participants were Chinese, and therefore, we used the BMI cut-off recommended by the World Health Organization for the Chinese population. Furthermore, more breast cancer studies have been conducted in Western countries than in China. Consequently, there may be some differences in our results from previous ones due to racial and demographic factors. (2) The limited number of cases in this study and the single-center study may lead to some bias in statistical results. Future large sample, multi-center, and prospective clinical studies are needed to further explore the influence pattern of BMI on the prognosis of female breast cancer, so as to provide evidence for early identification of breast cancer patients with high risk of poor prognosis. (3) Follow-up time was short and treatment options, such as endocrine therapy or chemotherapy, and dosage were not analyzed. (4) There was a lack of information known to predict the clinical outcome of breast cancer in women, such as longitudinal changes in BMI after breast cancer diagnosis, modifiable lifestyle such as smoking, alcohol, and consumption of physical exercise, etc. (5) In our study, BMI was not considered in relation to disease-free survival and overall survival of different types of breast cancer, so further investigation is necessary.

## 5 Conclusion

In conclusion, our single-institution retrospective study showed a significant association between BMI and prognostic outcome among breast cancer patients in less developed areas of northern China. We acknowledge that the present study had some limitations including the retrospective study design and lack of information. Despite these limitations, our results indicated that obesity might be a risk factor for BCSS among Chinese female breast cancer patients, especially in premenopausal women with HER2 overexpression or triple negative subtype.
